# Reimbursed medication adherence enhancing interventions in 12 european countries: Current state of the art and future challenges

**DOI:** 10.3389/fphar.2022.944829

**Published:** 2022-08-11

**Authors:** Przemysław Kardas, Martina Bago, Pilar Barnestein-Fonseca, Kristina Garuolienė, Anne Gerd Granas, João Gregório, Maja Ortner Hadžiabdić, Barbora Kostalova, Francisca Leiva-Fernández, Pawel Lewek, Katerina Mala-Ladova, Marie Paule Schneider, Job F. M. van Boven, Daisy Volmer, Ioli Ziampara, Tamás Ágh

**Affiliations:** ^1^ Medication Adherence Research Centre, Department of Family Medicine, Medical University of Lodz, Lodz, Poland; ^2^ Reference Center of Pharmacoepidemiology, Research and Teaching Department, Andrija Stampar Teaching Institute of Public Health, Zagreb, Croatia; ^3^ CUDECA Institute for Training and Research in Palliative Care, CUDECA Hospice Foundation, Málaga, Spain; Instituto de Investigación Biomédica de Málaga-IBIMA Group C08: Pharma Economy: Clinical and Economic Evaluation of Medication and Palliative Care, Málaga, Spain; ^4^ Pharmacy Center, Institute of Biomedical Science, Faculty of Medicine, Vilnius University, Vilnius, Lithuania; ^5^ Section for Pharmaceutics and Social Pharmacy, Department of Pharmacy, University of Oslo, Oslo, Norway; ^6^ Norwegian Centre for E-health Research, University Hospital of North Norway, Tromsø, Norway; ^7^ CBIOS – Universidade Lusófona’s Research Center for Biosciences and Health Technologies, Lisbon, Portugal; ^8^ Centre for Applied Pharmacy, Faculty of Pharmacy and Biochemistry, University of Zagreb, Zagreb, Croatia; ^9^ Department of Social and Clinical Pharmacy, Faculty of Pharmacy in Hradec Kralove, Charles University, Hradec Kralove, Czech Republic; ^10^ Multiprofessional Teaching Unit of Community and Family Care Primary Care District Málaga-Guadalhorce, Andalusian Health Service (SAS), Instituto de Investigación Biomédica de Málaga-IBIMA Group C08, Málaga, Spain; ^11^ School of Pharmaceutical Sciences, University of Geneva, Geneva, Switzerland; ^12^ Institute of Pharmaceutical Sciences of Western Switzerland, University of Geneva, Geneva, Switzerland; ^13^ Department of Clinical Pharmacy and Pharmacology, Medication Adherence Expertise Center of the Northern Netherlands (MAECON), University Medical Center Groningen, University of Groningen, Groningen, Netherlands; ^14^ Faculty of Medicine, Institute of Pharmacy, University of Tartu, Tartu, Estonia; ^15^ Health Insurance Organization, National Health Insurance System, Nicosia, Cyprus; ^16^ Syreon Research Institute, Budapest, Hungary; ^17^ Center for Health Technology Assessment and Pharmacoeconomic Research, University of Pécs, Pécs, Hungary

**Keywords:** medication adherence, non-adherence, persistence, interventions, Europe, reimbursement, drugs, healthcare systems

## Abstract

**Background:** Medication non-adherence jeopardises the effectiveness of chronic therapies and negatively affects financial sustainability of healthcare systems. Available medication adherence-enhancing interventions (MAEIs) are utilised infrequently, and even more rarely reimbursed. The aim of this paper was to review reimbursed MAEIs across selected European countries.

**Methods:** Data on reimbursed MAEIs were collected from European countries at the ENABLE Cost Action expert meeting in September 2021. The identified MAEIs were analysed and clustered according to their characteristics, direct vs. indirect relation to adherence, and the targeted adherence phase.

**Results:** Out of 12 contributing countries, 10 reported reimbursed MAEIs, 28 in total, of which 20 were identified as MAEIs targeting adherence directly. Reimbursed MAEIs were most often performed by either doctors (*n* = 6), nurses (*n* = 6), or pharmacists (*n* = 3). The most common types of MAEIs were education (*n* = 6), medication regimen management (*n* = 5), and adherence monitoring feedback (*n* = 4). Only seven reimbursed MAEIs were technology-mediated, whereas 11 addressed two interlinked phases of medication adherence, *i.e.*, implementation and persistence.

**Conclusion:** Our review highlights the scarcity of reimbursed MAEIs across the selected European countries, and calls for their more frequent use and reimbursement.

## Introduction

Non-adherence to medications is one of the major issues faced by European healthcare systems. A recent scenario illustrating the problem is the common reluctance to accept anti-Covid-19 immunisation. Contrary to expectations, effective protection against potentially fatal infections, as well as all disadvantages of lockdowns and frozen economies, are not motivating millions of European citizens to accept freely available, effective vaccines (Robinson et al., 2021).

However, looking ahead, at least equally important are the consequences of non-adherence to chronic pharmacotherapy. Noteworthy, the need for such therapies has been rising in Europe due to the rapid aging of the European population, and the related increase in the number of those affected by multimorbidity (Quinaz Romana et al., 2020). Owing to pharmacologic therapies, the life expectancy of patients can be prolonged and their quality of life may be improved. Unfortunately, these benefits of evidence-based medicine are jeopardised by non-adherence. This is particularly apparent in its high prevalence, which reaches 50% among patients on chronic treatment, as underpinned by the dedicated World Health Organization (WHO) report (World Health Organization, 2003). Negatively affecting both individual and public health, it also leads to profound societal and economic consequences, such as increased morbidity, mortality, and healthcare utilisation (Walsh et al., 2019). Indeed, it has been estimated that medication non-adherence is associated with nearly 200,000 deaths as well as with up to 125 billion of potentially preventable direct (*e.g.*, hospitalizations, waste of medication) and indirect (*e.g.*, work productivity losses) costs annually in the European Union (European Commission, 2022). Finally yet importantly, it generates huge amounts of additional workload for healthcare professionals (HCPs) due to inaccurate diagnoses, ineffective treatments, need for additional consultations, or fear of medications as examples. In consequence, medication non-adherence seriously reduces financial sustainability of European healthcare systems.

The WHO model identified five clusters of factors affecting medication adherence, *i.e.*, patient, condition, therapy, healthcare system, and socio-economic factors (World Health Organization, 2003). Within each of these dimensions, multiple factors are identifiable (Kardas et al., 2013). After half a century of research in this area, several medication adherence-enhancing interventions (MAEIs) have been designed, addressing individual or multiple of these factors. A single MAEI does not solve the non-adherence problem, however, currently available interventions targeting chronic conditions may improve both adherence and clinical outcomes (Nieuwlaat et al., 2014).

Nevertheless, available MAEIs are not fully used. Stakeholders, including HCPs, still seem to be inadequately informed about the prevalence and consequences of non-adherence as well as the availability of effective solutions. Therefore, HCPs tend to overestimate the level of adherence of their patients, and thus, have low motivation to implement relevant interventions in practice (Kardas et al., 2015; Clyne et al., 2016; Kardas et al., 2021a). Without public investments and promotional activities, current application of preventive and corrective approaches is still mainly limited to clinical trials, thereby not reaching patients in real-life settings (van Boven et al., 2016).

Consequently, in the last decades, there has been little improvement in adherence management (Zullig et al., 2018). Therefore, wider implementation of MAEIs is urgently needed. It can lead to a multiple-win scenario: while contributing to the sustainability of European healthcare systems, it can also help individual patients and public payers, and at the same time increase the scope of innovation of the pharmaceutical industry, and boost overall economies. These effects have recently been well-illustrated with a study addressing adherence management in five European countries, which proved that medication adherence can not only save patient lives but also save national healthcare system costs (Mennini et al., 2015).

Therefore, it is of particular interest to study the enabling mechanisms for the wide-scale implementation of effective MAEIs. One of the major ones is their reimbursement. However, current literature lacks information on this issue, which precludes cross-border benchmarking of effectiveness of various reimbursement models and taking lessons from good practices that have already been identified. This was one of the major stimuli to create a novel network of European researchers, launched in 2020 under the name of “European Network to Advance Best practices & technoLogy on medication adherencE” (ENABLE). The ENABLE is a COST Action, funded by the European Commission, which brings together researchers from 39 European countries and Israel. ENABLE aims to raise awareness of adherence enhancing solutions, foster and extend multidisciplinary knowledge on medication adherence at patient, treatment, and system levels; accelerate translation of this knowledge from producers to useful clinical application; and work collaboratively towards economically viable policy and implementation of adherence enhancing technology across different European healthcare systems (van Boven et al., 2021). ENABLE is composed of four cohesive Working Groups (WGs) among which WG3 aims to facilitate the implementation of MAEIs in European healthcare settings. To obtain this goal, ENABLE WG3 is going to review national healthcare systems as well as the reimbursement pathways for adherence-enhancing technologies across different European countries. This will further allow comparative analysis, and benchmarking of various reimbursement models. This is very important since currently only a few countries systematically monitor adherence, and international benchmarking is still not in place (Khan and Socha-Dietrich, 2018).

The need for such an activity is particularly pronounced now. Recent research undertaken by ENABLE showed that during the second wave of the COVID-19 pandemic, most of management of chronic conditions has been shifted towards remote care. However, none of the European countries was fully prepared to assure maintenance of chronic treatments, and the MAEIs are not routinely embedded into chronic conditions management cycles (Kardas et al., 2021b; Ágh et al., 2021).

This paper, developed as a result of the working meeting of ENABLE WG3, aims to review reimbursed MAEIs currently available and those planned to be implemented soon across selected European countries, and to critically assess them against the predefined criteria. We performed this study in order to fill this knowledge gap, and stimulate further introduction of verified MAEIs into European healthcare systems.

## Methodology

### Study design

A report from international expert round table, supplemented by consultation of national adherence experts across Europe.

### Setting and data collection process

An ENABLE WG3 expert meeting was held in Lodz, Poland, between 16 and 17 September 2021. It was devoted to a discussion on pan-European challenges and opportunities for reimbursement of MAEIs. ENABLE partners (including academics with medical or pharmaceutical backgrounds, HCPs, health economists and other stakeholders) were invited to take part in the meeting and provide a review of current medication adherence reimbursement scenarios in their countries in 2021. Participants from 13 European countries (Croatia, Cyprus, the Czech Republic, Estonia, Hungary, Lithuania, the Netherlands, Norway, Poland, Portugal, Romania, Spain, Switzerland) attended the meeting either personally, or remotely, and gave presentations on MAEIs available in their countries, using a predefined template. In their presentations, the authors were instructed to adopt a national or regional perspective, rather than more local one, *i.e.*, disregard the interventions run by single institution, single-centre initiatives, clinical trials or research projects.

Public discussion during the meeting was followed by an iterative process of fine-tuning of individual countries’ input, which took place remotely after the meeting in several rounds. It finally allowed collating a cohesive description of identified MAEIs, using the same operational definitions for their description and classification. The key elements of the standard framework, *i.e.*, the definition and classification of MAEIs, classification of adherence phases, and reimbursement status of MAEIs, are further described in the following sections.

### Definition and classification of medication adherence enhancing interventions

MAEIs have been broadly defined as any formalised activities taking place within, or in association with the healthcare system, that in any way could positively affect medication adherence at individual patient level.

MAEIs were further divided into those directly or indirectly addressing medication adherence, of which the former were supposed to have medication adherence enhancement as either the primary focus, or at least one of the targets of a complex program.

A general consensus regarding the taxonomy of medication adherence enhancing interventions has not been reached yet. Therefore, in order to group the identified MAEIs into clusters, we have reviewed current literature and selected relevant publications (Demonceau et al., 2013; Kini and Ho, 2018; Torres-Robles et al., 2018; Wiecek et al., 2019; Cross et al., 2020). MAEI items extracted from these publications have been compared and modified to create a transparent and complete set of MAEI types to be used across this study, as illustrated in Table 1.

**TABLE 1:** Medication adherence enhancing interventions’ taxonomy adopted for this study.

Medication adherence enhancing interventions
1. Medication regimen management
2. Educational
3. Behavioural
4. Socio-psycho-affective
5. Reminders (physical and technical)
6. Technical equipment for monitoring the disease and providing feedback on outcomes
7. Adherence monitoring feedback based
8. Incentives and rewards
9. Complex (combination of two and more interventions as described above)
10. Other

### Classification of adherence phases

According to the ABC Taxonomy (Vrijens et al., 2012), three phases of medication adherence continuum can be distinguished:

• Initiation–which occurs when the patient takes the first dose of a prescribed medication–being typically a binary event.
• Implementation–the extent to which the patient’s actual dosing corresponds to the prescribed dosing regimen, from initiation until the last dose is taken–being a longitudinal description of patient behaviour over time, *i.e.*, their dosing history.
• Discontinuation–which occurs when the patient stops implementing the prescribed medication–being typically a binary event. Consequently, persistence is defined as the time elapsing from initiation, until eventual treatment discontinuation (*i.e.*, time to event).

### Reimbursement of MAEIs

By “reimbursed MAEIs” we understood those subject to reimbursement by various organizations, such as public healthcare systems, governments, public or private insurance options, pharmaceutical companies, patient organizations, or others. However, interventions paid only through out-of-pocket by individual patients were not regarded as “reimbursed MAEIs.” In other words, by “reimbursed interventions” we understood only those which were not paid by patients.

## Results

Country updates on the MAEIs reimbursement status were provided by representatives from 12 European countries (i.e. Croatia, Cyprus, the Czech Republic, Estonia, Hungary, Lithuania, the Netherlands, Norway, Poland, Portugal, Spain, and Switzerland). In total, 32 reimbursed interventions were reported. These are numbered consecutively in the paragraphs below in braces, *e.g*., {7}, and described in details, along with interventions planned to be implemented in the near future.

### Croatia

There are no reimbursed MAEIs at any level of the healthcare system currently available in Croatia. Thus, implementation of this kind of interventions depends on the individual awareness of a particular HCP only. Reimbursed interventions indirectly targeting adherence are available at the primary care level, within the so-called Diagnostic Therapeutic Procedures (DTP) which are combinations of fee-for-service payments and performance bonuses for GPs. These are: {1} medication review for persons older than 65 years with three or more prescribed drugs aimed at increasing effectiveness and safety of the therapy (6.94 €) and {2} panels for chronic patients (*e.g*., diabetes, chronic obstructive pulmonary disease or hypertension) (0.67 €). These panels facilitate the work of GPs through systematic recording, monitoring in predefined time intervals and IT support for treatment of chronic patients and consequent actions to improve disease management, *e.g*., therapy modifications and/or patient education. The system alerts GPs in predefined time intervals of the need to monitor certain parameters for an individual patient with the aim of managing their chronic diseases. Monitoring of particular parameters (*e.g*., glycated haemoglobin, HbA1c, lipid profile, spirometry etc.) is additionally reimbursed. However, due to the shortage of medical doctors, overwhelming tasks they provide and low awareness of the adherence problem, adherence focused interventions are rarely employed in daily practice.

Future plans for the introduction of new reimbursed MAEIs are proposed in the National Recovery and Resilience Plan for Croatia 2021–2026, released in July 2021 (Vlada Republike Hrvatske, 2021). One of the planned investments is “Introduction of a system for monitoring the outcomes of treatment of outpatient chronic patients in community pharmacies.” As stated in the plan, using an appropriate software the pharmacist would record all important medical and pharmacological data related to the patient’s therapy (*e.g*., adherence, side effects, achievement of targets, *etc*.). All data on the outcomes of treatment of an individual patient will be transferred to a central information system and will be available for analysis to prescribers and insurers. The proposed pharmacists’ procedure will be structured into DTP and reimbursed by the Croatian Health Insurance Fund (CHIF). The estimated overall cost for initiation of the system is 574,056 €.

### Cyprus, Republic of

Prior to the National Health Insurance System (NHS), which was launched in 2019 and completed in 2020, the existence of two different subsystems (public and private sector), hindered the implementation of a universally endorsed adherence monitoring system. After the implementation of NHS, a record system collecting information about patients’ medication does exist. Electronic monitoring of adherence can be indirectly done by the system through the prescription issues and refill rates, limitation of repetitions of medical prescriptions (max. 6 months) and the number of packages dispensed (according to the defined daily doses, DDDs), and definition of a specific period during which the patient can take his/her medication. This monitoring includes structural assessment of the dispensation of several medicinal products prone to poor adherence, *e.g*., {3} valsartan and {4} deferasirox. The monitoring is organized by the Health Insurance Organisation (which is the competent authority for managing NHS) at the national statistics level and it does not involve direct interaction with patients.

Introduction of new reimbursed MAEI is planned for the future. Firstly, the Health Insurance Organisation, in collaboration with the University of Cyprus, is drafting a new research program, whose objective will be observation of patient adherence in dementia and cardiovascular diseases, through Patient Reported Outcomes (PROs) and medical assessment. Moreover, there are plans to enrich current programs of assessment of dispensing of several medicinal products by adding parameters enabling indirect estimation of patient adherence, such as the rate of hospitalization, or ferritin levels for deferasirox therapy.

### Czech Republic

Currently, several reimbursed MAEIs directly addressing adherence are available in the Czech Republic according to the list of reimbursed health interventions established by the Ministry of Health. The MAEIs comprise {5} an anti-asthmatic drug (Enerzair Breezhaler^®^) equipped with electronic sensor which sends reminders and registers the correct use of inhalations. Using this inhaler, the patients can voluntarily share their data with the attending physician in order to assess adherence. The drug is subject to prescription by a specialist (pneumologist, allergologist, immunologist), and the intervention has been reimbursed since May 2021, currently covering 100 patients only. Other reimbursed MAEIs directly targeting adherence include patient education provided by nurses, which should cover, among others, issues related to medication adherence: {6} in psychiatry (75 min, once a year), with elements of motivational interviewing; during the education procedure, the nurse uses teaching aids by which she demonstrates the importance of taking medication, or provides comprehensive written materials; {7} in diabetes mellitus, targeted education on the principles and practical skills to improve self-management of diabetes (50 min, six times a year). Similar MAEIs are provided by medical doctors: diabetologists provide targeted education on the principles and practical skills to improve self-management of diabetes, in the form of {8} a group session with max. six patients (30 min, once a year) or in the form of {9} an individual consultation (lasting for 40 min, four times a year). Medical doctors also provide {10} educational interviews with a patient/family, covering issues related to medication adherence (30 min, one time at new therapy initiation).

Two other reimbursed MAEIs available in the Czech Republic indirectly targeting adherence are: {11} education about inhalation technique, provided by nurses in chronic airways conditions (10 min, once a year, or in case of therapy change); and {12} complex assessment of the patient’s risk of drug-related problems, determining of patient pharmacotherapy rationalization plan, and verification of the effectiveness of the patient’s pharmacotherapy including education regarding the patient’s prescribed pharmacotherapy, provided by clinical pharmacists in inpatients, (15–20 min, once or twice per hospitalization) as well as in outpatients (15 min upon a medical doctor’s request).

Along with this, one non-reimbursed MAEI has been identified, *i.e.*, individual consultation in a community pharmacy about the patient’s pharmacotherapy, including medication adherence. Since 2020, patients, medical doctors, and pharmacists have been provided with access to the shared medication list with all issued ePrescriptions recorded and this may contribute to evaluation and support of medication adherence among these stakeholders. There is also a plan to introduce a new reimbursed MAEI, *i.e.*, individual counselling with the patient in community pharmacies, which is supposed to target the evaluation of medication and actual or potential drug-related problems, including medication non-adherence, and lead to relevant modifications of the pharmacotherapeutic regimen.

### Estonia

Several reimbursed MAEIs directly and indirectly targeting adherence are currently available in Estonia:

1. {13} Integrated drug-drug interaction database and clinical decision support system allowing GPs to assess the compatibility of the prescribed medicines with all currently used ones. When non-compatibility is identified, the physician decides whether to intervene, *e.g*., reduce the dose, *etc.* While potential interactions are identified early, it should prevent major drug-related problems and thus indirectly improve adherence. Pharmacists can use the same database to identify potential interactions between prescription and OTC medicines.
2. Specialised nurses - asthma nurses, diabetes nurses and mental health nurses - have independent (from physicians) appointments for outpatients: {14} The asthma nurse provides instructions on the use of medications and on disease control (25 min). If necessary, a second repeat visit is arranged to check the ability to use the medicine (up to 20 min). {15} A diabetes nurse teaches the patient how to use the glucometer and inject insulin. {16} At the appointment of the mental health nurse (regular visits up to 30 min), in addition to other topics, the administration of medicines under the supervision of the nurse is provided, drug information is shared, the treatment regimen is taught and the concentration of the medicine in the blood serum is monitored.

Along with these, two non-reimbursed MAEIs are currently available in Estonia:

1. Automated dose dispensing service - The service is provided by a private company for ambulatory and nursing home patients, however, in reality most of the clients are residents of nursing homes. Medicines that are needed for 2 months are dispensed in a plastic strip in daily units. The service includes an initial medication review by a pharmacist. The service (*i.e*., packaging) is paid by patients.^1^
2. Pharmacy-based reminder service for patients to renew prescriptions - it is a phone-based prescription renewal service. The patient has to give the pharmacist access to their e-prescriptions and authority to communicate with the prescriber on their behalf. Once there is a need for prescription renewal, the pharmacist will contact the doctor. In case the prescription cannot be renewed or there is a need for consultation, the pharmacist will inform the patient by phone before they run out of medicines. For the patient, the service is free of charge. The service is linked to one of the online pharmacies, so that medicines are directly delivered to the patient.^2^

Moreover, there are some plans for the introduction of new MAEIs. Namely, Medication Review (MR) service at community pharmacies, directly targeting adherence, is prepared. This service has been already piloted in Estonia and the results have been presented to the authorities. A service standard is being developed according to which the MR service is provided to patients with chronic conditions and five or more medications. The need for the service is assessed and the service is recommended to the patient by a general practitioner or a pharmacist, however, the patient can also apply for the service independently. In order to achieve the objectives of the service, it must take place at least twice in a row at the beginning. The overall frequency of the service could be twice a year (Tuula et al., 2021).

### Hungary

In the past few years, several MAEIs have been implemented in Hungary. In 2009, the National Health Insurance Fund (NEAK) established an indicator system for primary healthcare, which directly targets medication adherence (Kovács et al., 2019; NEAK, 2019). The system provides financial incentives for general practitioners (GPs) who reach the desired target values. Currently, 16 indicators are used to assess adult practices applied by GPs, and one of these directly evaluates medication adherence: {17} Indicator #7: the proportion of patients with myocardial infarction, coronary bypass, or percutaneous transluminal coronary angioplasty who filled prescriptions for beta-blockers at least 4 times in the previous 12 months. GPs are eligible for an extra payment if their patient achieves the goal value as set by the NEAK. However, this indicator system has only a negligible effect on the financing of primary care (3.8% in 2018) and thus it does not have a real motivational effect on everyday patient management (Rurik, 2019).

Another MAEI directly targeting adherence financed from public funds is the {18} “Three Generations for Health Program,” which was introduced in 2018 by the Ministry of Human Resources for consortiums of general medical practices^3^. As part of the program, GPs could get financial support for improving medication adherence of chronic patients aged 40–65 years by means of education and regular monitoring (1–3 months) of medication adherence (*e.g.*, with a standardized questionnaire such as the Morisky scale). The program was pre-financed and its financial accounting has been based on a fee-for-service model. Since 2018 the program has had two 1-year calls; the second was closed in 2021 and no new call has been announced so far.

In Hungary, there are also other MAEIs which are financed by pharma companies or other organizations. Among them, the one that directly targets adherence is the {19} free e-health application called HABITA^™^, which was introduced in the middle of 2021. It is a Hungarian language digital blood pressure and medication diary application for hypertensive patients with reminders to take medications and to refill prescriptions. Another example is {20} the “Be Educated and Empowered Patient” (BEEP) program which was launched in 2016. BEEP is an education program for organ transplanted patients organized by the Hungarian Transplant Federation thanks to various funds from different pharmaceutical companies and state grants^4^. The program primarily aims to improve the health literacy level and health behaviour of newly transplanted patients and thus it has an indirect effect on medication adherence as well.

In 2017, the National eHealth Infrastructure (EESZT)^5^ was established in Hungary. EESZT is a cloud-based, communication interface that connects healthcare providers (from 2020 including also private service providers) and pharmacies. The system contains all medical data uploaded after 1 November 2017 and transfers the health data (including medical records, prescription and refill data) of all patients to a central database. The system could greatly contribute to monitoring medication adherence; however, currently there is no reimbursed national initiative for this, nor any firm plans of introducing new reimbursed MAEIs in Hungary in the near future.

### Lithuania

In Lithuania, {21} GPs are able to hire another nurse whose employment is funded by the health insurance fund. These nurses monitor conditions of patients, including the use of medicines and, if necessary, provide them with advice, as well as refill prescriptions. However, only a few health care institutions have implemented such a practise so far.

Another intervention indirectly influencing adherence comes under the framework of the {22} Quality Guide of the Pharmacy Service which sets standards for the activities of community pharmacies, among the others a pharmacy care service aiming to teach patient how to use inhaled drugs. There is the rule book, how to teach patients to use inhaled drugs, approved by the Minister of Health^6^. The quality of service provision is monitored and evaluated through regular self-analysis. Pharmacists do not get any payment from health insurance for that service. The patients themselves have to pay out-of-pocket, therefore this service is not widely used.

The pharmaceutical policy document includes planned measures to implement IT systems to support monitoring medication adherence. However, the document neither provides clear indicators nor highlights dedicated activities.

### Netherlands

In the Netherlands, a mandatory multiple private insurer system exists. Basic health insurance coverage is mandatory and health insurers need to accept all people and provide the basic service package. Items to be reimbursed out of the basic service package (drugs, primary care, secondary care, *etc.*) are selected by central assessment of the Dutch Healthcare Institute (Zorginstituut Nederland, ZIN) based on their efficacy, necessity, practicality and cost-effectiveness.

Two types of adherence enhancing interventions, *i.e.*, drug-device combinations, and complex (behavioural) interventions, have different routes to reimbursement in the Netherlands, according to the universal criteria.

Drug-device combinations are devices integrated with a drug and dispensed at the same time. An example of such a solution is the {23} smart inhaler (Enerzair^®^ Breezhaler^®^) linked to mobile application which has been assessed by ZIN for maintenance treatment of asthma in adult patients, and found to be worth including in the Medication Reimbursement System (GVS) at List 1B, meaning free pricing by the manufacturer (not exceeding European averages). In principle, doctors prescribe it, however, patients receive automated reminders on their smartphones and do self-monitor themselves using the application. The results are available in the app and may be shared with the doctor during a consultation.

Complex behavioural MAEIs are reimbursed in other way: for this type of interventions a separate reimbursement code for healthcare providers/HCPs (pay per performance) can be applied for at the Dutch Healthcare authority (NZa) by stakeholders (*i.e.*, groups of healthcare professionals and/or individual insurers). An example of such a solution is an {24} adherence to asthma/COPD medication intervention that has a separate NZa coding (“NZa prestatie”). This intervention is provided by pharmacists. It entails identifying nonadherence, finding out reasons for nonadherence, providing adherence enhancing interventions and evaluating its outcomes. The price per performance of the “NZa prestatie” is negotiated between an individual HCP or chain of HCPs and a health insurance company. To get an “NZa prestatie” reimbursed, specific criteria need to be met, *e.g.*

1. A HCP (*e.g.*, a pharmacist) enhances adherence (*e.g.*, by motivational interviewing, counselling), makes changes regarding a medication and/or its dose based on available data.
2. A HCP discusses medication intake issues with the patient and how the medication should be used.
3. A HCP highlights the importance of persistence and finds concordance with the patient.
4. A HCP provides the patient with structure (*i.e.*, integration of medication intake with daily routine such as brushing teeth or walking the dog) to help them use the medication properly
5. The adherence is tracked by assessment of prescription refill/dispensing patterns in the future to see changes.
6. Interventions are registered in the patient’s record.

### Norway

Currently, pharmacies in Norway provide several reimbursed MAEIs:

{25} Multidose drug dispensing (MDD), established in 2006**,** is a form of adherence aid that provides patients with machine-dispensed medicines in disposable plastic bags, usually for 14 days (Josendal et al., 2021). The MDD bags are labelled with the patient’s name, drug names and the time the medicines should be taken. Tablets and capsules can be dispensed *via* MDD, while medicines such as mixtures, inhalators, topical formulations, *etc.*, are dispensed in their original packaging.
{26} Checking the inhaler technique - this service was officially launched in 2016 by the Minister of Health. Pharmacists check a patient’s inhalation technique and provide guidance on how to use the device correctly.
{27} New Medicine Service (established in 2018) is intended for patients with cardiovascular diseases who start a new cardiovascular drug. The intervention consists of two 15-min counselling sessions (in a pharmacy or by phone). The first session takes place 1–2 weeks after the patient collects the medicine at the pharmacy, and the second session after the next 3–5 weeks. The focus of this MAEI is: *1*) building good habits, *2*) gaining knowledge and understanding of the prescribed treatment, and *3*) practical problems and difficulties related to adherence to treatment. The efficacy of this intervention was tested in a randomised controlled trial, and was found effective (Hovland et al., 2020).

Moreover, a new reimbursed MAEIs directly targeting medication adherence in diabetes is to be launched soon under the name of “Medicine start–diabetes.”

### Poland

No reimbursed MAEIs are currently available in Poland. One of the few mechanisms that might be regarded as a non-reimbursed MAEI indirectly addressing medication adherence is generic substitution. According to the legally binding regulations, instead of more expensive drug prescribed for a patient, pharmacists are formally obliged to propose a more affordable equivalent. This could certainly remove one of the major barriers towards adherence. Unfortunately, a recent analysis proved that this mechanism is used extremely infrequently: substitution of original drugs with their generic equivalents was observed in less than 5% (Kardas et al., 2021c). It is noteworthy that the pharmacists are not incentivised to provide this service.

Another non-reimbursed MAEI indirectly addressing adherence is freely available national IT solution dedicated for healthcare professionals: on-line platform www.gabinet.gov.pl. It allows for management of various daily patient-oriented tasks, e.g. prescribing of e-prescriptions. The new functionality, provided recently, allows checking whether an individual prescription has been filled in. This functionality is based on the comparison of e-prescription and dispensation data, which both are recorded within nationwide eHealth systems secured by a dedicated governmental institution, eHealth Center (Polish: Centum e-Zdrowia, CeZ). Commercial office IT systems for doctors tend to adopt this functionality, as well, and often allow for control of the amount of medications prescribed, preventing excessive prescribing and informing on gaps in drug possession due to insufficient prescribing.

There are some plans for a new reimbursed MAEI, indirectly targeting adherence, due to the recently passed Polish Act on the Profession of Pharmacists (Dziennik Ustaw, 2021) which introduces pharmaceutical care that has not existed in Poland before. Among innovations codified by the Act, there is a new service planned within pharmaceutical care which will cover identification, management and prevention of drug problems in general. It will also include drug reviews^7^. Originally targeted towards polypharmacy and drug-drug interactions, it might be expected to support patient adherence as well.

### Portugal

Currently, there are no reimbursed interventions that directly target medication adherence available in Portugal. However, there are several interventions that target medication accessibility (thus indirectly helping medication adherence) that are reimbursed. One of these is {28} the “Operation Green Light,” which was launched with the COVID-19 pandemic. It ensures transfer of medicines dispensing exclusive for ambulatory hospital pharmacies to community pharmacies, without any costs for the patients. This intervention was reimbursed by a special program from a social care association–Associação Dignitude - Programa Abem, and the recipients of the reimbursement were providers/medicine distributors.^8^ However, pharmacists were not specifically paid for taking part in this intervention. This nationwide intervention is now undergoing evaluation to assess the possibility of its being maintained beyond the current pandemic context.

Moreover, it can be argued that the {29} co-payment system in place to facilitate the access to medicines at the community pharmacy is an indirect intervention regarding medication adherence.

Apart from maintaining this intervention in the future, there are no known plans to introduce any other intervention in the coming months. It is possible that pharmaceutical care consultations offered by community pharmacists’ may be re-introduced in the coming years and may be reimbursed, however, for now it remains a scenario only.

### Spain

At the national level, two major initiatives have been launched in Spain, having an indirect impact on adherence, *i.e*., the Electronic Prescription program and the Electronic Health Record System (EHRS). In 2013, the interoperable electronic prescription service of the National Health System (RESNS) was launched, allowing dispensation of medication prescribed in another autonomous community from any pharmacy, by electronic means. The only requirement was to present an individual health card^9^.

In recent years, several initiatives have been carried out in Spain to review the use and management of medicines where improving adherence to treatment is also particularly relevant. In 2016, the Plan for Treatment Adherence was launched as a collaborative work of scientific medical, pharmaceutical and nursing societies, patient representatives and other expert professionals, with the impulse of Big Pharma. The plan suggests facilitator activities (having adequate time-per-patient; reaching agreement with patients; individualising treatments) and establishes five main pathways to improve the level of patient adherence: raise awareness about the importance of adherence, establish a specific program with a system of information about it, simplify therapeutic regimens and increase patient self-management and empowerment^10^.

The Plan has been partially followed in different Spanish regions that have developed several programs aimed at reviewing the use and management of medications. These are multidisciplinary intervention programs where joint efforts of doctors, nurses and pharmacists aim to improve medication adherence. For example, in Andalusia, they currently work on designing a {30} medication review in complex patients with multiple chronic conditions and 15 or more medications prescribed for 180 days or more. In order to meet this goal, functionality of EHRS is used. The ultimate aim of this activity is a Personalized Action Plan (PAP) based on a comprehensive assessment of the patient’s health problems and current treatment, incorporating the pharmacological assessment. PAP involves the family doctor, the community nurse and the pharmacist in primary care.

One of the groups most involved in improving adherence in Spain are pharmacists. They are currently working on various strategies and new MAEIs. One of these strategies is the AdherenciaMED project, demonstrating the efficacy and effectiveness of the Therapeutic Adherence Service on health outcomes, at a clinical, economic and humanistic level (Gastelurrutia et al., 2020). Another approach adopted is the development of a Personalised Treatment Dosage Systems. However, no firm plans to introduce new MAEIs are known now.

### Switzerland

Although health service research grants in Switzerland have been supporting innovative medication adherence programs, MAEIs have not been yet a priority for the Swiss public authorities. Yet, a few MAEIs have been reimbursed in Switzerland for a long time, *e.g*., {31} the preparation of weekly pill-organizers either by pharmacists or nurses. This service targets polypharmacy patients on long-term conditions. For example, pharmacists are paid a fixed fee per week if the service is prescribed by the physician for patients taking at least three different long-term drugs (Hersberger and Messerli, 2016). Another intervention is {32} the Direct Observed Therapy (DOT) for any medication delivered in a community pharmacy and prescribed by a physician for patients encountering important adherence issues (*e.g.,* opioids, disulphiram, tuberculosis treatment, HIV treatment) (Hersberger and Messerli, 2016). Eventually, a new MAEI is being prepared based on the New Medicine Service implemented in the UK, where community pharmacists would be able to invoice two 10-min consultations for supporting a patient’s initiation of any new long-term treatment.

### Synthesis of results: Identified MAEIs

After careful consideration, out of the aforementioned 32 reported interventions, four were not accepted as satisfying the operational definitions of reimbursed MAEIs, *i.e.*

• monitoring of the dispensation of products likely to cause poor adherence (valsartan {3} and deferasirox {4}, both coming from Cyprus) – as these monitoring is organised at the high level and does not affect individual patients in any way, it rather serves national statistics;
• patient education on how to use inhaled drugs in compliance with the standards set by the Quality Guide of the Pharmacy Service, reported from Lithuania {22}: due to the fact that the patients themselves need to pay for this service, and it is not subject to reimbursement; and
• co-payment system in place to facilitate the access to medicines at the community pharmacies–reported from Portugal {29}, yet most probably, available in each and every European country. Undoubtedly, reimbursement of prescription drugs is an important enabler of drug access, on the other hand, this system is not specifically aimed to improve adherence, nor targeting individual patients.

**FIGURE 1:**
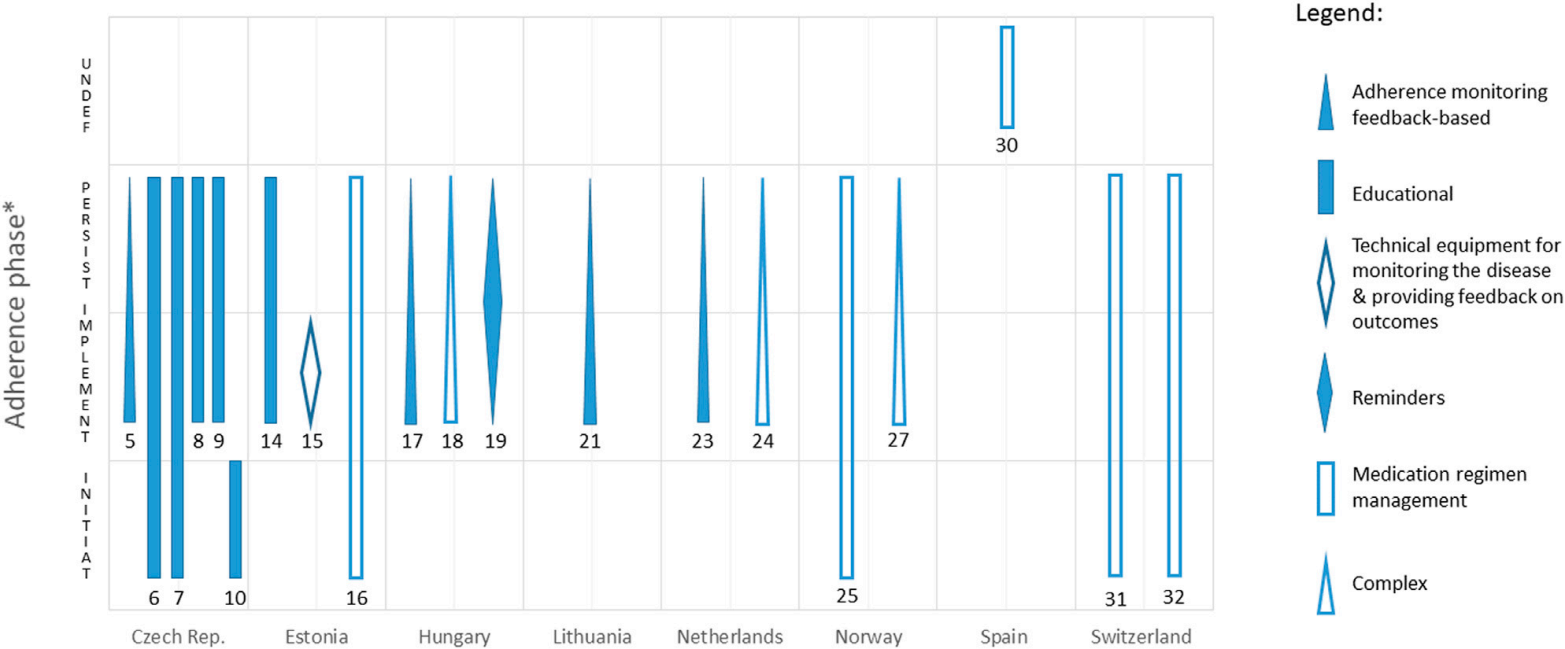
Adherence phases addressed by various reimbursed MAEIs directly targeting adherence, identified across analysed countries. * INITIAT, initiation; IMPLEMENT, implementation; PERSIST, persistence; UNDEF, undefined.

Thus, the final number of identified reimbursed MAEIs was 28. They are reported by 10 countries only (*see* Table 2). Out of that number, according to the adopted criteria, 20 interventions were identified as MAEIs directly targeting adherence, as listed in Table 3. The highest number of such interventions has been reported from the Czech Republic (6). MAEIs directly targeting adherence were performed most often by doctors (6), nurses (6), or pharmacists (3). The most common type of these interventions were the educational ones (6), followed by medication regimen management (5), adherence monitoring feedback based (4), and complex interventions (3). The least frequent interventions included the use of reminders (1) and technical equipment for monitoring the disease and providing feedback on outcomes (1). No reimbursed MAEI directly targeting adherence and representing the category of behavioural, socio-psycho-affective, or incentives and rewards has been reported. Only a minority of the reimbursed interventions (7 out of 20) were technology-mediated. Most of the interventions (11) addressed two interlinked phases of medication adherence continuum, *i.e.*, implementation and persistence, and 6 – all three phases, from the initiation to discontinuation (these belonging to either educational, or medication regimen management interventions only). Two MAEIs addressed only one adherence phase, and 1 could not be classified in terms of this dimension (for details, *see* Figure 1).

**TABLE 2:** Statistics of reimbursed MAEIs identified across 12 European countries.

Country	Currently employed interventions, *N*		Interventions planned in the future, *N*	
	Directly addressing medication adherence	Indirectly addressing medication adherence	Directly addressing medication adherence	Indirectly addressing medication adherence
Croatia	0	2	0	1
Cyprus, Republic of	0	0	3	0
Czech Republic	6	2	0	1
Estonia	3	1	1	0
Hungary	3	1	0	0
Lithuania	1	0	0	0
Netherlands	2	0	0	0
Norway	2	1	1	0
Poland	0	0	0	1
Portugal	0	1	0	0
Spain	1	0	0	0
Switzerland	2	0	1	0
TOTAL	20	8	6	3

**TABLE 3:** Detailed characteristics of reimbursed MAEIs directly targeting adherence, identified across 12 European countries.

Nr^a^	Country	Intervention	Who performs?	Type	Technology mediated?
5	Czech Republic	Inhaled drug (Enerzair Breezhaler^®^) for asthma therapy equipped with sensor and dedicated app	Patients	Adherence monitoring	Yes
6		Patient education in psychiatry; with elements of motivational interview	Nurse	Education	No
7		Patient education in diabetes, covering issues related to medication adherence	Nurse	Education	No
8		Patient education (group sessions), covering issues related to medication adherence	Doctor (diabetologist)	Education	No
9		Patient education (individual consultation), covering issues related to medication adherence	Doctor (diabetologist)	Education	No
10		Educational interview with a patient/family member covering issues related to medication adherence, provided at therapy initiation	Doctor	Education	No
14	Estonia	Patient education in asthma, covering issues related to medication adherence	Nurse	Education	No
15		Patient education in diabetes, covering issues related to disease self-monitoring with glucometer and medication adherence	Nurse	Technical equipment for monitoring the disease and providing feedback on outcomes	No
16		Directly observed therapy of mental conditions, accompanied by drug concentration monitoring	Nurse	Medication regimen management	No
17	Hungary	Primary care performance indicator: the proportion of patients with myocardial infarction, coronary bypass, or percutaneous transluminal coronary angioplasty who filled prescriptions for beta-blockers at least four times in the previous 12 months	Doctor (GP)	Adherence monitoring	Yes
18		Patient education and regular monitoring of medication adherence by GPs in chronic patients aged 40–65 years under the “Three Generations for Health Program”	Doctor (GP)	Complex	No
19		Mobile application for hypertensives providing blood pressure and medication diary with reminders to take medications and to refill prescriptions - HABITA^™^ e-health application	Patient	Reminders	Yes
21	Lithuania	Monitoring patients’ conditions including the use of medicines	Nurse (hired by GPs or Primary Health care institution)	Adherence monitoring	No
23	Netherlands	Inhaled drug (Enerzair Breezhaler^®^) equipped with sensor and app for asthma therapy	Patients	Adherence monitoring	Yes
24		Complex behavioural intervention targeting adherence to asthma/COPD medication	Pharmacists	Complex	No
25	Norway	Multidose drug dispensing providing patients with machine-dispensed medicines	Pharmacists (in collaboration with GPs and nurses)	Medication regimen management	Yes
27		New Medicine Service for patients with cardiovascular diseases	Pharmacists	Complex	No
30	Spain	Medication review in complex patients with multiple chronic conditions and 15 or more medications prescribed	Pharmacists, GPs and nurses in collaboration	Medication regimen management	Yes
31	Switzerland	Preparation of weekly pill organizers	Pharmacists or nurses	Medication regimen management	Yes
32		Direct Observed Therapy for any patient encountering important adherence issues	Pharmacists	Medication regimen management	No

a Numbers refer to consecutive numbers ascribed to interventions across the country related paragraphs, appearing in braces; GP, general practitioner.

Characteristics of eight identified reimbursed MAEIs indirectly targeting adherence (Table 4) was similar to MAEIs directly addressing adherence: they were most often provided by doctors (three out of eight), represented the class of educational, or medication regimen management interventions (three in both cases). MAEIs indirectly targeting adherence were supported by technology even less frequently than those targeting adherence directly.

**TABLE 4:** Detailed characteristics of reimbursed MAEIs indirectly targeting adherence, identified across 12 European countries.

Nr^a^	Country	Intervention	Who performs?	Type	Technology mediated?
1	Croatia	Medication review for persons aged over 65 years with three or more prescribed drugs, aiming to increase effectiveness and safety of the therapy	GP	Complex	No
2		Panels for chronic patients (e.g. diabetes, COPD or hypertension) involving monitoring of chronic disease and relevant interventions to improve disease management, e.g. therapy modifications and/or patient education	GP	Complex	No
11	Czech Republic	Patient education about inhalation technique in chronic airways conditions	Nurse	Education	No
12		Assessment of patient risk of drug-related problems, determining his/her pharmacotherapy rationalization plan, provided to inpatients and outpatients	Clinical pharmacists	Medication regimen management	No
13	Estonia	Drug-drug interaction database and clinical decision support system, providing compatibility of the prescribed medicines with all existing medicines	GP	Medication regimen management	Yes
20	Hungary	“Be Educated and Empowered Patient” program aimed at improving health literacy and health behaviour in newly transplanted patients	Patient organisation	Education	No
26	Norway	Patient education in asthma, including checking inhaler use technique	Pharmacists	Education	No
28	Portugal	“Operation Green Light” allowing dispensation of medicines previously available exclusively in hospital pharmacies in community pharmacies	Pharmacists	Medication regimen management	No

a Numbers refer to consecutive numbers ascribed to interventions across the country related paragraphs, appearing in braces; COPD, chronic obstructive pulmonary disease; GP, general practitioner.

## Discussion

Our review identified several reimbursed MAEIs available across the European countries discussed. Noteworthy, these interventions have diverse characteristics. There are interventions directly as well as indirectly targeting adherence. Moreover, they target different phases of the adherence continuum, most often the implementation and persistence. Finally, they are provided by various stakeholders (*i.e*., doctors, nurses, pharmacists, patients). They employ various targets and methodologies, from educational ones, up to the use of technical equipment for monitoring the treatment and the disease.

On the other hand, only a minority (seven out of 20) of identified reimbursed MAEIs directly addressing adherence were technology-mediated, whereas most of them were based on interpersonal collaborative skills, *e.g*., patient education, or directly observed therapy executed by HCPs. Several classes of MAEIs were not reported from the reviewed countries. Moreover, two reviewed countries (*i.e*., Cyprus and Poland) did not report any reimbursed MAEIs or any plans for their upcoming introduction. It all, undoubtedly, points to the potential underuse of various available MAEIs, and particularly, the technology-mediated ones.

Along with these principal findings, we have identified an urgent need for setting uniform standards in terms of MAEI terminology and taxonomy. The operational definitions accepted by us in this review were inclusive by purpose. This, however, leads to certain freedom of interpretation, and thus, the differences between two types of MAEIs were not always clear, requiring one or more rounds of iterative discussions to obtain an internal consensus on their classification. For the same reasons of inclusiveness, we have adopted a “patient perspective” for the issue of MAEIs reimbursement, accepting all those which were free for patients as the reimbursed ones. Among such interventions, some are simply made available, by providing HCPs with relevant tools, standards, or guidelines in order to execute them. However, other MAEIs are subject to dedicated payment to those who execute them, perhaps much more completely fulfilling an intuitive definition of “reimbursed interventions.” This definitely requires further studies which should collect more detailed economic data.

Currently, we clustered MAEIs based on their characteristics and/or the techniques employed. However, we found that the reimbursement of identified MAEIs comes from both private and public resources, which may be an important denominator of the interventions for further classification. Perhaps, MAEIs could be further grouped in relevant clusters according to the determinants of adherence being tackled by each individual intervention.

Wider implementation of effective MAEIs is of utmost importance as medication non-adherence places a significant cost burden on healthcare systems. According to the results of a recent systematic literature review, the annual cost of non-adherence per person ranged from $949 to $44,190 across 14 disease groups (Cutler et al., 2018). Therefore, even if healthcare systems need to assign special funds for reimbursement of MAEIs, it is reasonable to consider such expenditures. Improving adherence is shown to be an economically viable treatment option for patients with various chronic conditions. Many studies confirmed cost-effectiveness of various MAEIs. For example, a systematic review of interventions adopted in asthma management found that all of the assessed MAEIs were cost-effective considering the increased adherence rate, improved clinical effectiveness and the reduced costs of asthma care (Khaw et al., 2021). Similarly, the cost of a pharmacist-led medication adherence management service for chronic patients was estimated to be €27.33 ± 0.43 per patient for 6 months, which resulted in an incremental cost-utility ratio (ICUR) of €2,086.30/QALY, thus proving that the intervention was time-consuming, yet cost-effective (Valverde-Merino et al., 2021). In another study, despite increased drug costs, better medication adherence was assessed as cost-effective in chronic conditions. The average cost-benefit ratios from adherence for the four conditions examined varied from 1:3.8 for hyperlipidaemia to 1:13.5 for hypertension. Hence, one extra 1 USD spent on medications for adherent patients with typical chronic conditions (congestive heart failure, high blood pressure, diabetes and hyperlipidaemia) can generate between 3 and 13 USD in savings on emergency department visits and hospitalisations (Roebuck et al., 2011).

Several interventions have been shown to effectively improve long-term medication adherence. Therefore, another question is which one to adopt in a particular scenario. Perhaps, in order to optimize cost-effectiveness of these interventions, and thus improve the probability of positive reimbursement decisions, and their more frequent implementation, it is necessary to adopt targeted interventions, tailored to the needs of a specific patient. (van Boven et al., 2016). Thus, the choice of an individual MAEI is important. A recent meta-analysis found that the effect of MAEIs seems to be disease-specific: the most effective ones differed across various clinical conditions (*e.g*., educational and technical MAEIs resulted in a major effect in terms of improving medication adherence in patients with HIV, circulatory system and metabolic diseases, whereas attitudinal MAEIs presented a more powerful effect on musculoskeletal and mental disorders) (Torres-Robles et al., 2018).

Similarly, there is evidence proving the beneficial role of novel MAEIs, based on eHealth, over medication adherence (Jeminiwa et al., 2019; Aardoom et al., 2020). However, not all technology-mediated MAEIs or eHealth interventions are of equal effectiveness (Mistry et al., 2015; Schulte et al., 2021). Thus, currently there is still some uncertainty regarding the timing, duration, intensity, and specific types of eHealth-enabled MAEIs that could be most effectively implemented by health care providers. The more widespread use of design science, implementation science or other co-creation methodologies which provide a high degree of interaction between all the relevant stakeholders and context, to design and implement eHealth-enabled MAEIs is a possible solution moving forward (De Geest et al., 2020; Gregório et al., 2021).

Unfortunately, despite all the evidence, clearly pointing at health, societal and economic benefits of improved medication adherence, healthcare systems do not take much action to support medication adherence in patients with chronic conditions. In consequence, we are still far from a widespread use of MAEIs. The scenario was neither changed by the call to address medication non-adherence, clearly stated by the seminal WHO report in 2003 (World Health Organization, 2003), nor the policy recommendations for promoting medication adherence produced within the dedicated European research collaboration - the ABC project^11^. A survey administered in 2017 to the authorities of member countries of the Organization for Economic Co-operation and Development (OECD) found that in most of them non-adherence is still not considered a priority on the national policy agenda. Interventions to enhance or support medication adherence are not well coordinated. They do not constitute part of a larger strategic policy programme either. Most countries dvelop guidelines, frameworks, and financial incentives for primary care physicians or interprofessional team to improve quality of care for patients with chronic diseases (Khan and Socha-Dietrich, 2018). However, no specific physician- and few interprofessional-delivered adherence interventions were reported by the participating countries.

In particular, reimbursement of MAEIs has not received much attention until now. Interestingly enough, this issue was neither tackled in the extensive Cochrane review on interventions for enhancing medication adherence (Nieuwlaat et al., 2014) nor in the WHO report on chronic diseases management in Europe (Nolte et al., 2014).

A limited success of many adherence-promoting programs may result from the lack of inclusion of and limited support for engaged stakeholders. Meanwhile, financial incentives play an important role in improving adherence (Khan and Socha-Dietrich, 2018). A study that examined physicians’ preferences found that doctors’ willingness to implement adherence-promoting programs in daily practice was determined by the time commitment to carry out the program (34.8% importance), reimbursement (33.3%), and validation status of the program (23.7%) (Müller et al., 2020). A better understanding of the impact of interprofessional collaborations to support medication adherence throughout the patient’s therapeutic care journey is needed. It may indicate the added value of the reimbursed MAEIs which provide a win-win opportunity to both patients, and healthcare providers.

**FIGURE 2:**
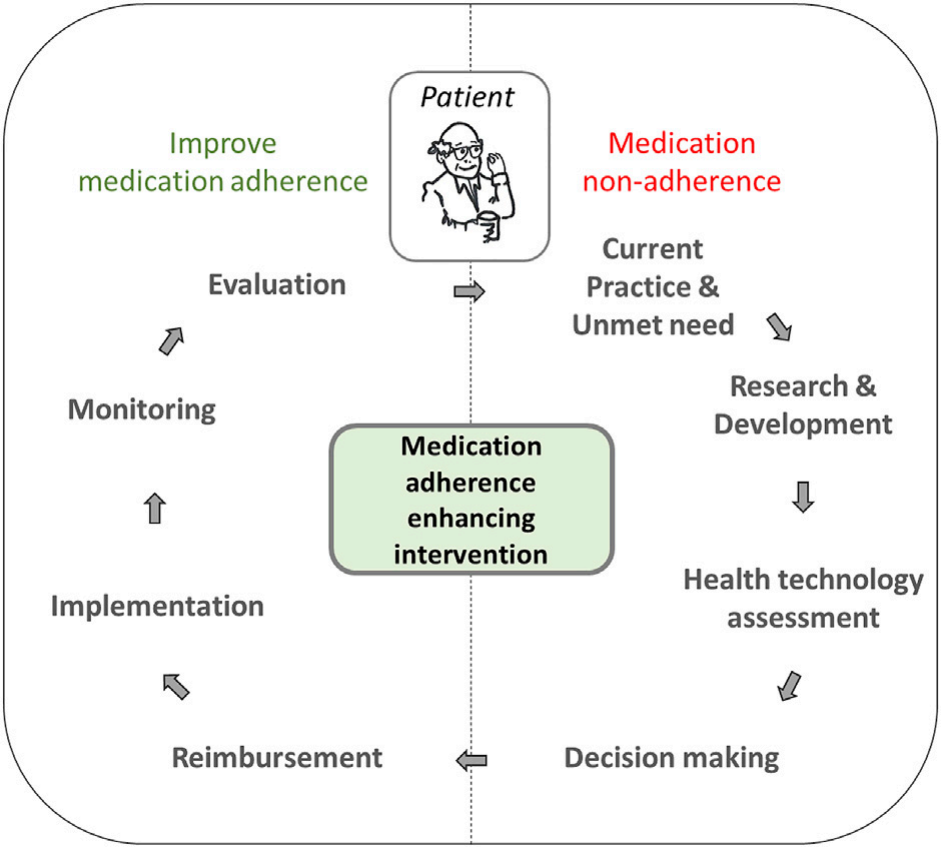
Scheme of iterative medication adherence-bettering process, involving reimbursement of medication adherence enhancing interventions.

The results of our review undoubtedly point to the scarcity of reimbursed MAEIs across the studied European countries. Despite large availability of effective, and cost-effective interventions, the plans for implementation of new reimbursed MAEIs in these countries in the nearest future are not impressive either. Perhaps, the major lesson that one should learn from that scenario is that obviously, the time is high for a change. It does not only involve MAEIs which should be more widely implemented across Europe. There also exists an urgent need to create a well-balanced, evidence-based process of iterative improvement of medication adherence, as proposed in Figure 2. The patient plays an active part in the process as a member of the interprofessional team based on shared decision making. A careful approach to design, implementation and evaluation of reimbursement mechanisms is the key component of the process.

Along with scarcity of interventions targeting adherence identified in the participating countries, we have also found major gaps in the current approach towards MAEIs, which together creates an urgent need for adoption of a uniform approach to address the issue. Therefore, as a result of the ENABLE WG3 Lodz meeting, and this in-depth review, we have designed European level recommendations which call for:

1. A clear and uniform taxonomy for MAEIs.
2. Formulation of minimum standards for the clinical evaluation and health technology assessment of MAEIs.
3. Transferability of MAEIs from one healthcare system to another.
4. Recommendations on reimbursement pathways for the different types of MAEIs.
5. Standard evaluation procedures for the reimbursement and implementation of MAEIs embedded into national healthcare systems.

Medication adherence is accepted as a measure of care quality (Seabury et al., 2019). Taking this into consideration, international experts emphasized that adherence to treatment is a right of chronic patients. Thus they urged European healthcare systems to finance programs at optimising adherence and stimulating collaboration of other parties such as industry and national governments (van Boven et al., 2017). A practical use of such an approach has been proposed by a dedicated OECD report, which suggests a broadly-used medication adherence as a measure for performance-based reimbursement contracts. In its conclusion, the OECD report identifies four enablers of the improved medication adherence at the system level, these being acknowledging, informing, incentivising, and steering/supporting. (Khan and Socha-Dietrich, 2018).

Our results should be considered in the light of certain limitations. First of all, it should be kept in mind that the method of collecting information on the availability of MAEIs, which was based on personal knowledge of the authors, may not entirely reflect all the interventions available in a particular country. Similarly, various professional backgrounds of the authors may add bias to the scope of the reporting. The authors made every effort (*e.g*., by holding external consultations) in order to provide a full picture. Nevertheless, despite the iterative process of provision, and fine-tuning of the country feedback, certain MAEIs might have been underreported. For instance, some education programs comprising support for medication adherence and integrated in the standard clinical practice might have been considered as reimbursed MAIEs by some countries (*e.g*., the Czech Republic), however, not by other (*e.g*., Switzerland). It is also uncertain if any official list of reimbursed MAIEs is available in each country, which makes the situation even more difficult.

Moreover, lack of generally accepted taxonomy of MAEIs led to the use of the operational definitions, which not always were very precise, thus living the space for subjectivism in MAEI classification. In the future, this obstacle could be overcome by the use of a predefined taxonomy.

Finally, only infrequently the economic details of particular MAEIs were fully available, thus counteracting fair benchmarking of the identified interventions. In future studies, this dimension is worth special attention.

On the other hand, this study has a number of strengths. According to the authors’ knowledge, the ENABLE WG3 working meeting which took place in Lodz, Poland, between 16 and 17 September 2021 was the first European expert meeting collecting information on the reimbursed MAEIs in a systematic way. Therefore, in future research, the findings of the meeting may serve as a source of inspiration for considering certain interventions to be MAEIs, and broadening the scope of reporting. Also, we believe that our operational definitions may stimulate further work on MAEIs taxonomy.

In conclusion, our review highlights the scarcity of reimbursed MAEIs across the selected European countries. However, we hope that our work, being the first review of its kind on reimbursed MAEIs, paves the way to further studies, which by advancing benchmarking of MAEIs, may stimulate their more common visibility, adoption, and reimbursement across European countries.

## Data Availability

The original contributions presented in the study are included in the article further inquiries can be directed to the corresponding author/s

## Footnotes

1 Automated Dose Dispensing. Available online: https://www.apotheka.ee/teenused/ravimite-personaalne-pakendamine/ (Accessed 17.02.2022).

2 Apotheka Reminder Service. Available online: https://www.apotheka.ee/teenused/apotheka-meeldetuletaja (Accessed 17.02.2022).

3 Three Generations for Health Program. Available online: https://okfo.gov.hu/3g-program (accessed on 03 Oct 2021).

4 Be Educated and Empowered Patient (BEEP) program. Available online: http://www.trapilap.hu/ (accessed on 03 Oct 2021).

5 National eHealth Infrastructure (EESZT). Available online: https://e-egeszsegugy.gov.hu/web/eeszt-information-portal/home (accessed on 03 Oct 2021).

6 Lietuvos Respublikos sveikatos apsaugos ministerija. ĮSAKYMAS DĖL FARMACINĖS RŪPYBOS PASLAUGOS ĮKVEPIAMUOSIUS VAISTINIUS PREPARATUS VARTOJANTIEMS PACIENTAMS TEIKIMO TVARKOS APRAŠO PATVIRTINIMO. TAR, 2016-06-20, Nr. 17250. Available online: https://e-seimas.lrs.lt/portal/legalAct/lt/TAD/2b4114c0372211e6a222b0cd86c2adfc?jfwid=https://e-seimas.lrs.lt/portal/legalAct/lt/TAD/2b4114c0372211e6a222b0cd86c2adfc?jfwid (accessed on 17.02.2022).[Not Available in CrossRef]

7 Ministerstwo Zdrowia. Raport opieka farmaceutyczna. Kompleksowa analiza procesu wdrożenia. Warszawa, 2020. Available online: https://www.gov.pl/web/zdrowie/opieka-farmaceutyczna–-raport. (accesses on 17.02.2022)[Not Available in CrossRef]

8 Associação Dignitude. Emergência ABEM: COVID 19 [Internet]. 2020. Available online: https://abem.dignitude.org/emergencia-abem-covid-19/ (accessed on 06.10.2021.)

9 Ministerio de Sanidad, Consumo y Bienestar Social - Profesionales - Receta Electrónica. Available online: https://www.mscbs.gob.es/profesionales/recetaElectronicaSNS/home.htm (accessed on 25.10.2021.)

10 Farmaindustria. Plan de adherencia al tratamiento. Available online: https://www.farmaindustria.es/adherencia/ (accessed on 17.02.2022)[Not Available in CrossRef]

11 Final report of the ABC Project. Available online: http://abcproject.eu/img/ABC%20Final.pdf (accessed on 17.02.2022).
